# Acute Cholangitis Secondary to Surgical Clip Migration 18 Years After Cholecystectomy: A Case Report

**DOI:** 10.7759/cureus.21975

**Published:** 2022-02-07

**Authors:** Izyan N Mohammad, Chee F Chong, Vui H Chong

**Affiliations:** 1 Medicine, Raja Isteri Pengiran Anak Saleha (RIPAS) Hospital, Bandar Seri Begawan, BRN; 2 Surgery, Raja Isteri Pengiran Anak Saleha (RIPAS) Hospital, Bandar Seri Begawan, BRN

**Keywords:** complications, cholecystectomy, surgical clip migration, cholangitis, obstructive jaundice

## Abstract

Gallstone disease is a common condition and reason for consultation and hospitalizations. The standard of care is laparoscopic cholecystectomy. Early complications include bile duct injury and retained stone, and chronic complications include bile duct stricture and clip migration. It is important for clinicians to be aware of such complications as they can occur long after surgery. We report an interesting case of clip migration resulting in acute cholangitis, 18 years after laparoscopic cholecystectomy and review the literature on this interesting phenomenon of a commonly performed surgery. The diagnosis of clip migration in our case was suspected on abdominal radiograph and confirmed on endoscopic stone extraction.

## Introduction

Gallstone disease is one of the most common gastrointestinal conditions and represents a common cause for the hospital admission. Patients may present with biliary colic, cholecystitis, pancreatitis, or obstructive jaundice [1]. The standard of care for symptomatic gallstones disease is laparoscopic cholecystectomy [2] and is one of the most common surgeries carried out. Laparoscopic cholecystectomy is generally safe but can be associated with complications such as retained stones or bile duct injury [2]. We report an interesting case of acute cholangitis secondary to a metal clip migration 18 years after laparoscopic cholecystectomy and review of the literature on this interesting complication of a commonly performed surgery.

## Case presentation

A 46-year-old female presented with severe epigastric pain lasting several hours and fever. She described the pain as intermittent and colicky, coming in waves for the past 24 hours. This had worsened on the day of the presentation. She also had mild nausea but no vomiting. On examination, she was febrile (38.0^o^ Celsius), jaundiced, and had mild tenderness in the epigastric area. She was otherwise hemodynamically stable (pulse rate 85 per minute, blood pressure 135/84 mmHg).

Her past medical history included hyperlipidemia, thalassemia minor, and laparoscopic cholecystectomy 18 years previously. Three weeks prior to her latest presentation, she presented to the emergency department with upper abdominal pain, and an ultrasound scan (USS) of the abdomen showed post-cholecystectomy status with a non-dilated common bile duct (CBD) 5 mm. Blood investigations including complete blood count, liver profiles, and amylase were normal. Antispasmodic medication was given to the patient for symptomatic treatment.

**Figure 1:**
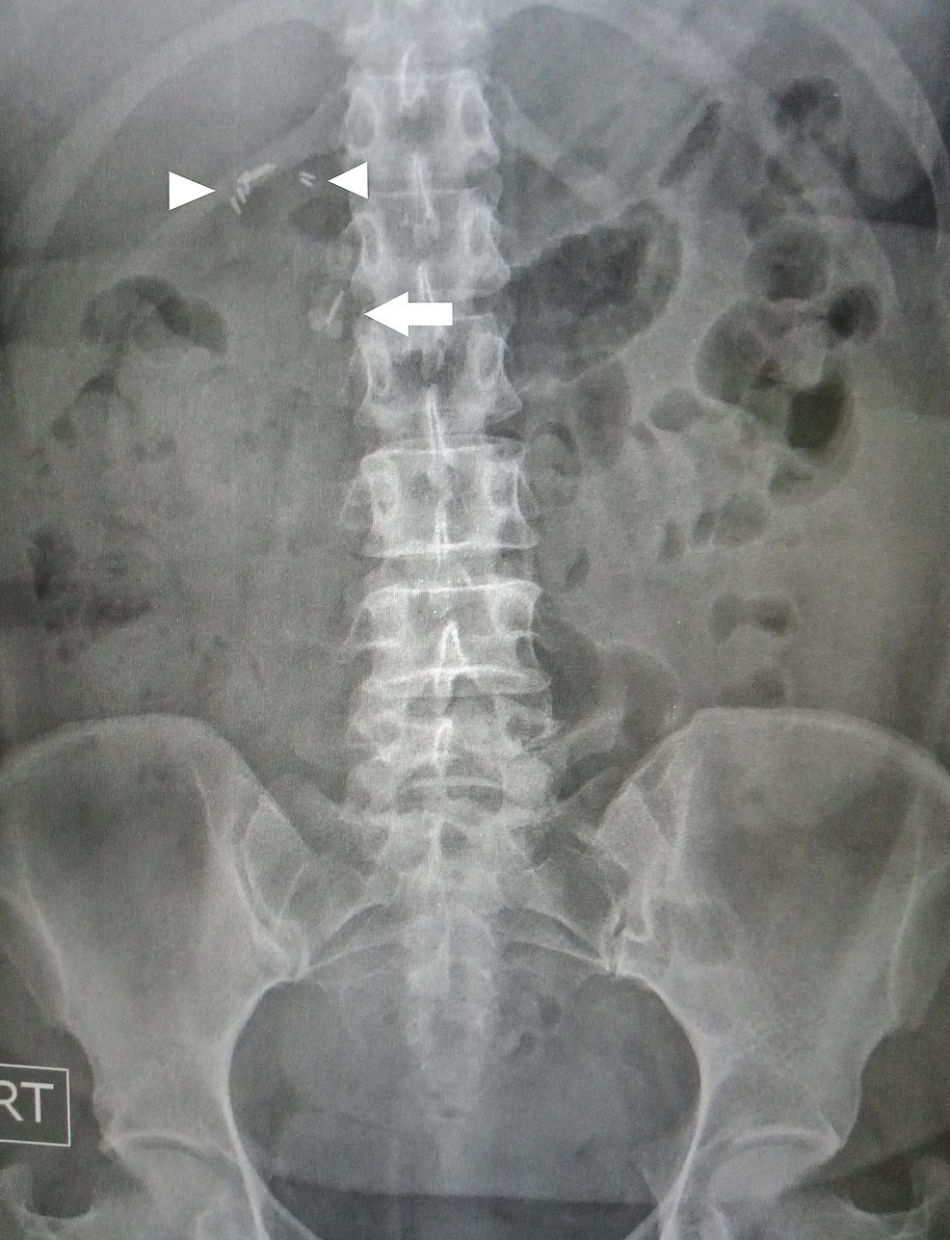
Abdominal X-ray showing surgical clips at level of T12/L1 (arrowheads) and another clip at L1/L2 (arrow). Abdominal X-ray showing surgical clips at level of T12/L1 (arrowheads) and another clip at L1/L2 (arrow).

Her latest blood investigations showed cholestatic liver profiles; total bilirubin 71 umol/L (normal range 5.1-20.5), alanine aminotransferase 145 U/L (normal range 1-54), alkaline phosphatase 254 U/L (normal range, 38-126), and gamma-glutamyltransferase 188 U/L (normal range 7-64), and elevated inflammatory markers: C-reactive protein 12.6mg/dL (normal range 0.00-0.75) and white blood cell (WBC) count of 16800 (normal range 4.2-12.6 x 10^3^/uL). Serum amylase was normal. The abdominal radiograph showed several surgical clips from a previous cholecystectomy. Interestingly, one clip was noted to be located away from the main bunch (Figure 1) raising the suspicion of clip migration. A repeat USS showed that the CBD was more dilated (9 mm) than the previous scan. No stones or cause of dilatation could be appreciated due to bowel gas.

**Figure 2:**
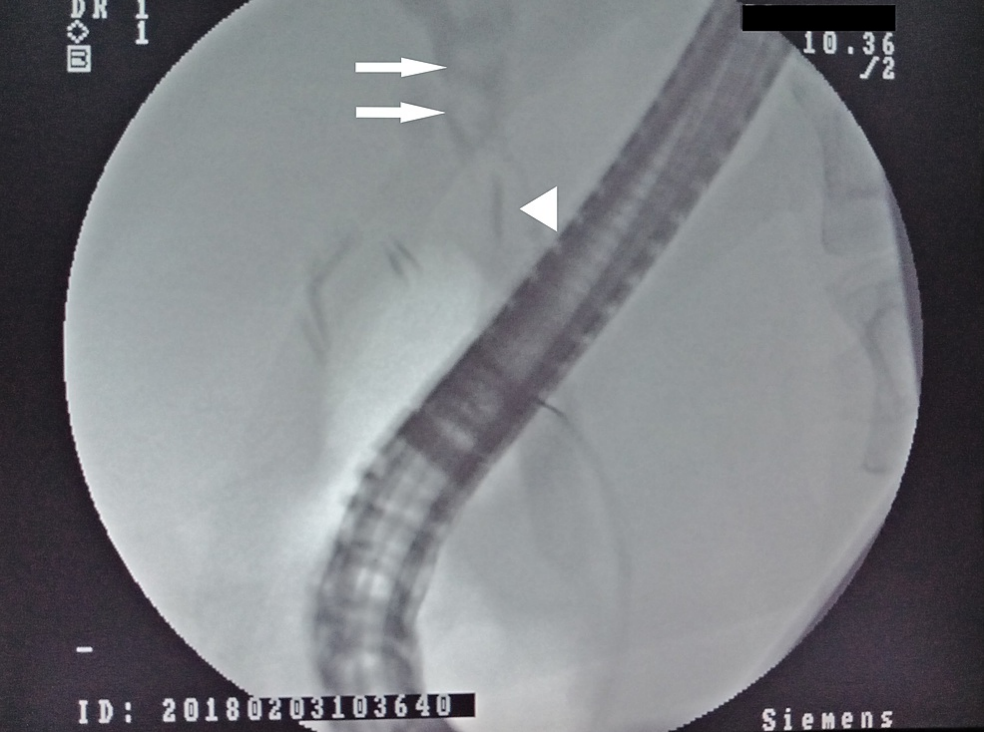
ERCP cholangiogram showing surgical clips, several filling defects (bile duct stone indicated by arrows), and one with the surgical clip at the center (arrowhead). ERCP: endoscopic retrograde cholangiopancreatography

**Figure 3:**
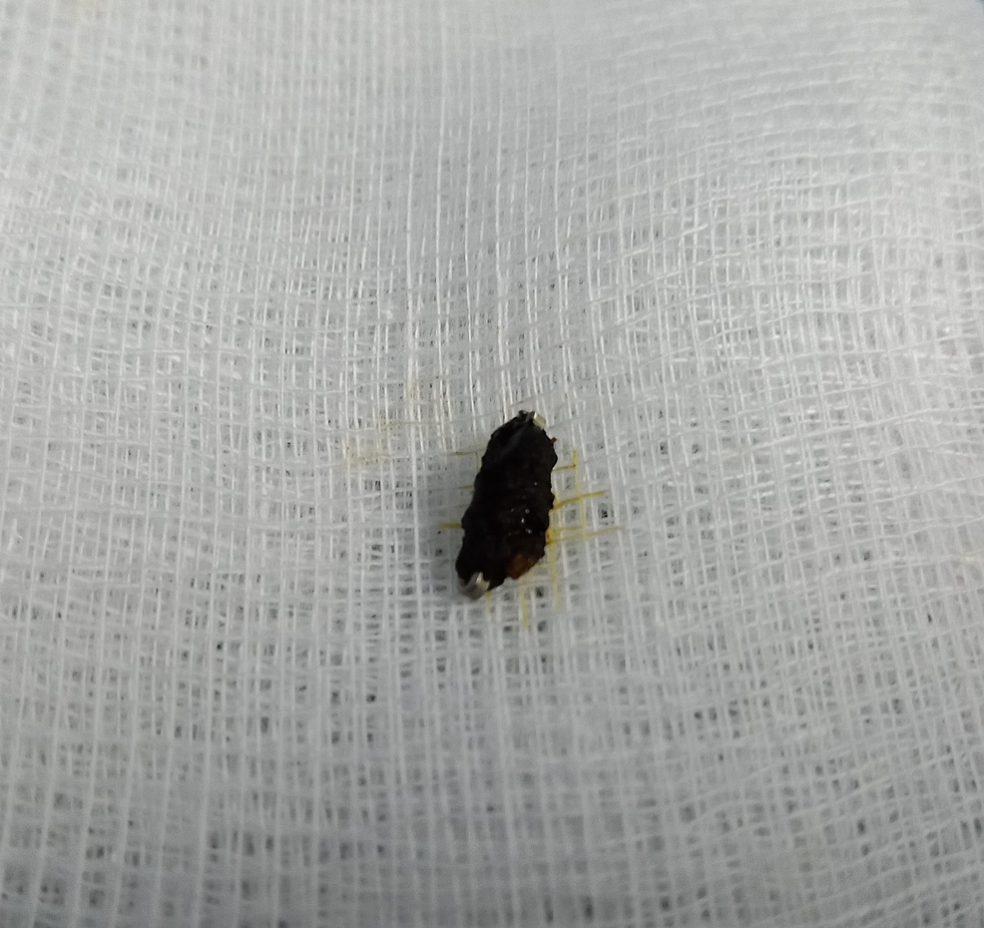
Extracted fragment of black pigment stone with surgical clip at the center.

A diagnosis of acute cholangitis was made and she was started on an intravenous antibiotic (Cefaperazone 1000 mg twice daily), intravenous fluid, and as required intramuscular analgesia. Blood cultures did not isolate any organism. In view of her clinical presentation, blood investigations, and dilated duct on USS, she was counseled for endoscopic retrograde cholangiopancreatography (ERCP). Cholangiogram revealed multiple filling defects (stones), one with a dense linear dense opacity (surgical clip) located at the center (Figure 2) consistent with stone formed over a migrated surgical clip. After endoscopic sphincterotomy, balloon trawling of the biliary system extracted several black pigment stones including the one with the clip. The stone with the surgical clip was extracted and was retrieved using a basket for inspection. On fragmenting the stone, the clip could be easily seen (Figure 3). The patient’s symptoms improved thereafter and was discharged several days later to complete a week of antibiotics. She had remained well without any symptoms during the two years of follow-up.

## Discussion

Postcholecystectomy clip migration (PCCM) was first reported in 1979 after open cholecystectomy and in 1992 after laparoscopic cholecystectomy [3,4]. As the number of cholecystectomies performed increased over the years [5], the number of cases of PCCM reported has also increased and continued to be reported [6-8]. A literature search of publications in English and non-English has yielded mainly case reports or case series with only two publications that had analyzed and summarized the published cases. Chong et al. identified 80 cases of PCCM into the bile duct published between 1979 and 2007, of which 69 were included in the review [5]. Pang et al. identified 53 publications published between 1997 to 2017 which included 64 cases [6]. Of these, 49 (76.6%) were PCCM into the bile duct [6]. Ng et al. in their review identified 54 publications between 1993 and 2019 [7]. This study did not provide summary analyses of the cases. 

Literature search for publications between 2018 and 2021 using the terms ‘bile duct’ + ‘clip migration’ and ‘cholecystectomy’ + ‘clip migration’ in PubMed and Google Scholar, followed by a manual search of references identified 36 publications in the English and non-English literature between 2018 and 2021. In total, 53 cases of PCCM into the biliary tree were reported, indicating an increase in the number of cases reported compared to the previous periods. This increase may either be due to increased awareness and reporting, or an actual increase in the incidence of clip migration. Of 36 publications, 12 publications were from 2020 and 2021 [7-18]. The characteristic details of the reported cases between the three-time periods are shown in Table 1. Compared to the two earlier periods, it appears that in the later period (publications between 2018-2021), there is no female predominance, older age at presentations, and longer median (and mean) interval between cholecystectomies and presentations.

**Table 1:** Findings of the two large reviews and latest literature search. LC: laparoscopic cholecystectomy, LCBD: laparoscopic cholecystectomy and bile duct exploration, OC: open cholecystectomy, OLT: orthoptic liver transplantation, ERCP: endoscopic retrograde cholangiopancreatography

	Chong et al (2010) [5]	Pang et al (2019) [6]	Latest review
Number cases (period)	N = 69 (1978-2007)	N = 64 (1997-2017)	N= 53 (2018-2021)
Gender			
Female	42 (60.9%)	35 (54.6%)	26 (49.1%)
Male	26 (37.7%)	26 (40.6%)	26 (49.1%)
Not stated	1 (1.4%)	3 (4.7%)	1 (1.8%)
Median age (yrs)	60 (31-88)	59.75 (31-93)	65 (35 - 90)
Initial operation			
LC	47 (68.1%)	43 (67.1%)	37 (69.8%)
LCBD	Not stated	15 (23.4%)	11 (20.8%)
OC	25 (36.2%)	4 (6.2%)	4 (7.5%)
OC+OLT	1 (2.1%)	1 (1.6%)	0 (0%)
Not stated	Not stated	1 (1.6%)	1 (1.9%)
Duration from surgery to presentation	Median: 26 months	Median: 24 months	Median: 49 months
	Mean: not reported	Mean: 55.4 months	Mean: 86.3 months
	Range: 11 days to 20 yrs	Range 1 day to 20 years	Range: 1 month to 35 yrs
Symptoms reported			
Pain	58 (84.1%)	46 (71.8%)	41 (77.4%)
Jaundice	53 (76.8%)	30 (46.9%)	25 (47.2%)
Fever	22 (31.9%)	19 (29.7%)	21 (39.6%)
Nausea/vomiting	18 (26.1%)	18 (28.1%)	7 (13.2%)
Chills	Not stated	5 (7.8%)	1 (1.9%)
Asymptomatic	Not stated	12 (18.8%)	9 (17.0%)
Admitting diagnosis			
Obstructive jaundice	26 (37.7%)	Not stated	13 (24.5%)
Cholangitis	19 (27.5%)	Not stated	18 (33.9%)
Biliary colic	13 (18.8%)	Not stated	12 (22.6%)
Acute pancreatitis	6 (8.7%)	Not stated	1 (1.9%)
Incidental finding	3 (4.3%)	Not stated	9 (17.0%)
Abnormal liver profiles only	2 (2.8%)	Not stated	1 (1.9%)
Migrated lip on radiography	1 (1.4%)	Not stated	Not stated
Location of clip migrations			
Common bile duct	69 (100%)	49 (76.6%)	53 (100%)
Duodenum	0 (0%)	4 (6.3%)	0 (0%)
T-tube	0 (0%)	10 (15.6%)	0 (0%)
Gallbladder remnant	0 (0%)	1 (1.6%)	0 (0%)
Median number of migrated clips	1 (range 1- 6)	1 (range 1-4)	1 (range 1-6)
Type of clips			
Metal	Mostly metal	Not reported	35 (66.0%)
Absorbable	Not reported	Not reported	3 (5.7%)
Hem-o-Lok	Not reported	Not reported	15 (28.3%)
Management			
ERCP (Successful clearance)	53 (77.0%)	35 (54.7%)	37 (69.8%)
ERCP (failed clearance needing surgery)	Not reported	6 (9.4%)	2 (3.8%)
Surgery	14 (20.2%)	13 (21.3%)	7 (13.2%)
Choledochoscopic clearance	14 (20.2%)	12 (18.8%)	1 (1.9%)
Others	1 (1.4%)	4 (6.4%)	3 (5.7%)

The underlying pathogenesis and process of clip migration is unknown and is likely to be a complex and multifactorial situation. It is believed that continuous intra-abdominal pressure exerted on the surgical clips can lead to movement and over time, in some cases result in subsequent migration, usually into hollow viscera including the bile duct following the path of least resistance [5]. The speed of the movement is likely to depend on other factors. Risks for surgical clip migration are multifactorial and can be categorized into surgical and patient factors [5]. Surgical factors include inadequate technique and placement of too many surgical clips (either due to difficult surgery or poor techniques or experience). Ideally, only two clips should be left in place; one each for the cystic duct and cystic duct vessel. Placement and transecting of cystic duct too close to the cystic and bile duct junction has been postulated to be a factor [19]. External compression from the external structures on the cystic duct remnant can lead to envagination of the cystic duct stump and clip into the bile duct [20]. Over time, this can lead to clip migration. Patient factors include congenital anatomical anomalies or distorted anatomy either from previous surgery or inflammatory or fibrotic processes from previous cholecystitis contribute to difficult surgery, increasing the risk for bile duct injuries and placement of too many clips [5]. Active inflammatory processes from bile leak, biloma formation, and ongoing infection can lead to poor healing and breakdown contributing to clip dislodgement and migration. Alternatively, the clip may have been accidentally introduced into the biliary tree or placed with part of the clip inside the duct, a form of bile duct injury. The exposed part of the clip acts as a nidus for stone formation and over time, the concretion enlarges contributes to the process of inward migration. Once migration is complete, the stone falls into the biliary tree and eventually causes biliary obstruction [5]. 

Clinical presentations are similar to presentations of gallstones disease. Common symptoms include recurrent upper abdominal pain, fever, and jaundice (Table 1). Presentations with pancreatitis have also been reported. In severe cases, patients may present with acute cholangitis and septic shock. Surgical clip migrations have been reported to occur between one day and 35 years post-cholecystectomy [7], with a median of around two years post-cholecystectomy [5,6]. Early clip migration can be due to the inadvertent introduction of clips into the bile duct during surgery. Typically, when this happens or when clip migration occurs early, there may not be enough time for concretion accumulation to result in stone formation.

Evaluation is the same as for biliary stone disease with USS as the initial investigation followed by computed tomography (CT) or magnetic resonance cholangio-pancreatography (MRCP) if required. However, metallic clips as in our case can be easily visualized with plain abdominal radiography, and diagnosis can be made even before intervention and extraction of the clip-induced stone. However, non-metallic clips such as Hem-o-Lok are now more increasingly being used, clip migration may not be diagnosed until ERCP or surgery. This is reflected by the increasing number of cases of PCCM due to non-metallic clips [7].

The management of migrated clip and associated complications is the same as per choledocholithiasis; symptom control, antibiotic for infection such as cholangitis, and stone clearance. ERCP is the preferred option with a high successful clearance rate with surgery or percutaneous transhepatic clearance as the alternate option. 

PCCM is an interesting phenomenon that can occur soon to many years after cholecystectomy. Given that gallstones disease is a common condition and that current management practices are standard and safe, the number of laparoscopic cholecystectomies carried out annually will continue to increase. Therefore, the number of PCCM will likely increase and clinicians should be aware of this interesting complication. 

## Conclusions

This interesting case serves to highlight to all clinicians to be aware of this unusual and interesting complication of cholecystectomy that can occur many years after surgery. Although PCCM is a rare complication, clinicians should consider this possibility in patients who have had a cholecystectomy, especially if abdominal radiographs reveal abnormally located surgical clips.
